# Determinants of survival after first relapse of acute lymphoblastic leukemia: a Children’s Oncology Group study

**DOI:** 10.1038/s41375-024-02395-4

**Published:** 2024-09-11

**Authors:** Susan R. Rheingold, Deepa Bhojwani, Lingyun Ji, Xinxin Xu, Meenakshi Devidas, John A. Kairalla, Mary Shago, Nyla A. Heerema, Andrew J. Carroll, Heather Breidenbach, Michael Borowitz, Brent L. Wood, Anne L. Angiolillo, Barbara L. Asselin, W. Paul Bowman, Patrick Brown, ZoAnn E. Dreyer, Kimberly P. Dunsmore, Joanne M. Hilden, Eric Larsen, Kelly Maloney, Yousif Matloub, Leonard A. Mattano, Stuart S. Winter, Lia Gore, Naomi J. Winick, William L. Carroll, Stephen P. Hunger, Elizabeth A. Raetz, Mignon L. Loh

**Affiliations:** 1 Department of Pediatrics and the Center for Childhood Cancer Research, Children’s Hospital of Philadelphia, Perelman School of Medicine, University of Pennsylvania, Philadelphia, PA USA; 2 Division of Pediatric Hematology-Oncology, Children’s Hospital Los Angeles, Norris Comprehensive Cancer Center and Keck School of Medicine, University of Southern California, Los Angeles, CA USA; 3 Department of Population and Public Health Sciences, Keck School of Medicine, University of Southern California, Los Angeles, CA USA; 4 Children’s Oncology Group, Monrovia, CA USA; 5 Department of Global Pediatric Medicine, St Jude Children’s Research Hospital, Memphis, TN USA; 6 Department of Biostatistics, University of Florida, Gainesville, FL USA; 7 Department of Laboratory Medicine and Pathobiology, The Hospital for Sick Children, University of Toronto, Toronto, ON Canada; 8 Department of Pathology, The Ohio State University, Columbus, OH USA; 9 Department of Genetics, University of Alabama at Birmingham, Birmingham, AL USA; 10 The Hospital for Sick Children, Toronto, ON Canada; 11 Department of Pathology and Oncology, Johns Hopkins Medical Institutions, Baltimore, MD USA; 12 Department of Pathology and Laboratory Medicine, Childrens Hospital Los Angeles, Keck School of Medicine, University of Southern California, Los Angeles, CA USA; 13 Servier Pharmaceuticals, Boston, MA USA; 14 Department of Pediatrics, Golisano Children’s Hospital, Wilmot Cancer Center at University of Rochester Medical Center, Rochester, New York, NY USA; 15 Cook Children’s Medical Center, Fort Worth, TX USA; 16 Bristol Myers Squibb, Princeton, NJ USA; 17 Department of Pediatrics, Section of Hematology/Oncology, Texas Children’s Hospital, Baylor College of Medicine, Houston, TX USA; 18 Department of Pediatrics, University of Virginia School of Medicine, Charlottesville, VA USA; 19 Department of Pediatrics, University of Colorado School of Medicine and Center for Cancer and Blood Disorders, Children’s Hospital Colorado, Aurora, CO USA; 20 Department of Pediatrics, Maine Medical Center, Portland, ME USA; 21 Department of Pediatrics, Case Western Reserve University, Cleveland, OH USA; 22 HARP Pharma Consulting, Mystic, CT USA; 23 Cancer and Blood Disorders Program, Children’s Minnesota, Minneapolis, MN USA; 24 Department of Pediatrics, University of Texas Southwestern Medical Center, Childrens Health, Dallas, TX USA; 25 Perlmutter Cancer Center, Department of Pediatrics, NYU Grossman School of Medicine, New York, NY USA; 26 Department of Pediatrics, Seattle Children’s Hospital, Seattle, WA USA

**Keywords:** Risk factors, Acute lymphocytic leukaemia

## Abstract

Limited prognostic factors have been associated with overall survival (OS) post-relapse in childhood Acute Lymphoblastic Leukemia (ALL). Patients enrolled on 12 Children’s Oncology Group frontline ALL trials (1996–2014) were analyzed to assess for additional prognostic factors associated with OS post-relapse. Among 16,115 patients, 2053 (12.7%) relapsed. Relapse rates were similar for B-ALL (12.5%) and T-ALL (11.2%) while higher for infants (34.2%). Approximately 50% of B-ALL relapses occurred late (≥36 months) and 72.5% involved the marrow. Conversely, 64.8% of T-ALL relapses occurred early (<18 months) and 47.1% involved the central nervous system. The 5-year OS post-relapse for the entire cohort was 48.9 ± 1.2%; B-ALL:52.5 ± 1.3%, T-ALL:35.5 ± 3.3%, and infant ALL:21.5 ± 3.9%. OS varied by early, intermediate and late time-to-relapse; 25.8 ± 2.4%, 49.5 ± 2.2%, and 66.4 ± 1.8% respectively for B-ALL and 29.8 ± 3.9%, 33.3 ± 7.6%, 58 ± 9.8% for T-ALL. Patients with *ETV6::RUNX1* or Trisomy 4 + 10 had median time-to-relapse of 43 months and higher OS post-relapse 74.4 ± 3.1% and 70.2 ± 3.6%, respectively. Patients with hypodiploidy, *KMT2A*-rearrangement, and *TCF3::PBX1* had short median time-to-relapse (12.5-18 months) and poor OS post-relapse (14.2 ± 6.1%, 31.9 ± 7.7%, 36.8 ± 6.6%). Site-of-relapse varied by cytogenetic subtype. This large dataset provided the opportunity to identify risk factors for OS post-relapse to inform trial design and highlight populations with dismal outcomes post-relapse.

## Introduction

Acute lymphoblastic leukemia (ALL) is the most common childhood malignancy. With improved understanding of leukemia biology and the application of risk-directed therapy, 5-year event-free survival (EFS) for children in developed countries with newly diagnosed ALL is currently 85–90% [1–6]. However, for patients who relapse, outcome remains poor with limited progress made over the past three decades [7, 8].

During initial therapy, several well-established prognostic factors such as age, presenting white blood cell (WBC) count, central nervous system (CNS) involvement, cytogenetic and molecular subtype, and end-induction measurable residual disease (MRD) inform risk-directed therapy [9]. However, other than immunophenotype (B versus T), time-to-relapse, site of relapse, and MRD post re-induction, similar prognostic indicators have not been established for post-relapse outcomes [7, 10–14]. Smaller relapse studies suggest that patients with unfavorable cytogenetics or older age have worse outcomes but their role in risk stratification post-relapse is limited [11].

Children’s Oncology Group (COG) investigators previously reported overall survival (OS) after first relapse in children with ALL enrolled on an older group of non-overlapping frontline clinical trials between 1988 and 2002 [7]. Of 9585 patients on ten trials during this time period, 1961 (20.5%) relapsed. Five-year OS post-relapse for the entire cohort was 36%, similar to reports from other pediatric groups [10, 11]. In addition to time-to and site-of relapse, age at initial diagnosis, sex, and presence of CNS disease at diagnosis significantly influenced outcomes. However, due to limited immunophenotypic and cytogenetic data, detailed subgroup analyses were not possible.

In this report, we review the largest cohort to date of infant, pediatric, adolescent, and young adult patients with ALL enrolled on more contemporary COG trials. We identified additional clinical, cytogenetic, and biological risk factors that could enhance trial design and improve risk stratification algorithms for relapsed ALL.

## Patients and methods

### Patients and clinical trials

Patients with newly diagnosed B- or T-ALL enrolled on 12 frontline COG clinical trials from June 1996 - July 2014 were included in this study (*n* = 16,115; Table 1). Patients or guardians provided informed consent for trial participation. Patients <22 years were eligible for trials before 2004 after which eligibility expanded to patients <31 years. Trial details and 5-year EFS rates are listed in Table 1. For analysis of post-relapse survival, only patients with relapse as a first event were included. Patients with induction failure as defined by individual protocols (1.6%), death as first event (2.6%) and second malignant neoplasm as first event (0.96%) of 16,115 were excluded from the analysis. Data collected at first relapse included time from date of initial diagnosis to date of relapse (time-to-relapse), relapse site, and survival information. Data on subsequent therapy post-relapse were limited and therefore not addressed. Sex assigned at birth, race and ethnicity were per individual institutional submission.

**Table 1:** COG frontline trials included in this report.

Trial [reference]	Patient Type	Accrual dates	Risk group	*N* eligible	5-year EFS ± SE	No. of Relapses^a^ (% per protocol)	Time-to-relapse^b^ in months median (range)	Median follow-up in months from diagnosis^c^	Median follow-up in months from relapse^d^
CCG 1991 [15]	B-ALL and T-ALL *B-ALL* *T-ALL*	06/2000-01/2005	NCI Standard Risk	3026 2933 93	88.9 ± 0.6% 89.2 ± 0.6% 81.3 ± 4.1%	289 (9.6%) 276 (9.4%) 13 (14.0%)	32.5 (3.1–177.6) 33.5 (3.1–177.6) 16.1 (5.2–40.9)	138 138.8 122.2	92.5 92.5 91
POG 9404 [16]	T-ALL	06/1996-08/2001		363	74.0 ± 2.3%	56 (15.4%)	12.5 (1.1–132.5)	124.6	115.7
POG 9407 [17]	Infants (B-ALL)^e^	03/2001-06/2006		128	45.0 ± 4.5%	45 (35.2%)	13.2 (5.1–52.9)	82.9	60.4
POG 9904 [18]	B-ALL	04/2000-04/2005	9900 Low Risk	830	91.4 ± 1.0%	85 (10.2%)	43.0 (11.1–162.8)	126.6	81.5
POG 9905 [18]	B-ALL	05/2000-04/2005	9900 Standard Risk	1055	78.4 ± 1.3%	245 (23.2%)	31.1 (2.2–186.0)	87	49.9
POG 9906 [19]	B-ALL	05/2000-04/2003	9900 High Risk	272	62.1 ± 3.0%	102 (37.5%)	25.2 (1.7–118.1)	132.8	85.1
COG AALL0232 [20]	B-ALL	01/2004-01/2011	NCI High Risk	3104	75.1 ± 0.8%	490 (15.8%)	33.4 (2.6–139.5)	123.7	71.3
COG AALL0331 [21]	B-ALL	04/2005-05/2010	NCI Standard Risk	5307	89.6 ± 0.4%	471 (8.9%)	39.6 (2.1–169.4)	122.2	66.9
COG AALL0434 [22]	T-ALL	01/2007-07/2014		1563	83.8 ± 0.9%	158 (10.1%)	14.0 (0.3–113.8)	105.1	61.1
COG AALL0631 [23]	Infants (B-ALL)^e^	01/2008-06/2014		197	50.0 ± 3.6%	66 (33.5%)	14.5 (3.4–57.5)	97	70.8
COG AALL07P4 [24]	B-ALL	10/2008-12/2010	NCI High Risk	166	77.4 ± 3.3%	29 (17.5%)	30.9 (5.2–70.0)	120.2	65
COG AALL08P1 [25]	B-ALL	04/2009-01/2011	NCI High Risk	104	73.0 ± 4.4%	17 (16.3%)	25.5 (6.5–89.1)	134	89.9
TOTAL				16115	83.5 ± 0.3%	2053 (12.7%)	31.0 (0.3–186.0)	121.7	70.5

*ALL* Acute Lymphoblastic Leukemia, *CCG* Children’s Cancer Group, *COG* Children’s Oncology Group, *EFS* Event-free Survival, *NCI* National Cancer Institute, *POG* Pediatric Oncology Group, *SE* Standard Error.

^a^Not including induction deaths, treatment failures, or second malignant neoplasms.

^b^Only among relapsed ALL patients (time in months).

^c^Median follow-up time (time in months) from initial diagnosis among relapsed patients who were alive as of the last follow-up date.

^d^Median follow-up time (time in months) from relapse diagnosis among relapsed patients who were alive as of the last follow-up date.

^e^Only infants with B-ALL (93% of all enrolled patients on POG9407 and AALL0631) are included in this report.

The COG risk stratification algorithms used for patient eligibility are described in each trial publication [15–25]. Infants <1 year of age with B-ALL, treated on dedicated infant ALL trials, define the “Infant ALL” cohort in this report. Patients with B-ALL ≥ 1 year at diagnosis define the “B-ALL” cohort. Patients with T-ALL ≥ 1 year at diagnosis define the “T-ALL” cohort. All but 93 patients ≥1 year at diagnosis with T-ALL were treated on dedicated T-ALL trials. Outcomes post relapse in patients with Down Syndrome were reported separately [26].

### Definition of relapse

Patients who achieved a morphologic remission with <5% blasts and clearance of extramedullary disease with frontline therapy who then developed disease recurrence were defined as having a relapse. Bone marrow relapse was defined by ≥25% morphologic blasts or ≥5% blasts with concomitant extramedullary relapse, CNS relapse by CNS 3 status (≥5 WBC/microliter cerebrospinal fluid, with blasts on cytospin) or clinical signs of CNS leukemia. Isolated extramedullary recurrence had to be proven by biopsy. Relapses that occurred <18 months from diagnosis were considered “early”, 18- <36 months “intermediate”, and ≥36 months “late”.

### MRD determination and definitions

MRD was determined by centralized COG flow cytometry laboratories at the end of the 4-week Induction (EOI) therapy. On the CCG and POG trials MRD was done on a research basis and did not affect risk stratification but on the COG (AALL) trials, MRD was used in risk stratification and treatment decisions and are described in each trial publication [20–25]). For the majority of patients sensitivity was determined to 10^−4^, but in 87 patients data was only available to 10^−3^.

### Cytogenetics and molecular subtypes

From 1996 to 2003, karyotype data from 12 reference cytogenetics laboratories (Pediatric Oncology Group) or from more than 100 institutional cytogenetic laboratories (Children’s Cancer Group) was collected and reviewed by members of their central cytogenetics committees. The requirement for fluorescence in situ hybridization (FISH) studies for chromosomes 4, 10, and 17 was added for patients on COG trials in 2003 and was initially performed in COG central or approved local laboratories (2003 to 2006) and later (2007 to 2014) exclusively in COG approved local cytogenetic laboratories. Testing for *ETV6::RUNX1*, *BCR::ABL1*, *TCF3::PBX1* and *KMT2A* rearrangements was done by reverse transcriptase polymerase chain reaction (RT-PCR) or FISH in one of two central reference laboratories (2003 to 2006) or by FISH in COG approved local laboratories (2007 to 2014). Ascertainment of iAMP21 may have been incomplete prior to 2007. Standard karyotype analysis was performed in COG-approved local laboratories. All FISH and karyotype data from local laboratories were centrally reviewed. Patients found to have a *BCR::ABL1* translocation during Induction therapy would be removed from protocol therapy on the eligible trials in this analysis and, if available, offered enrollment in dedicated Ph+ ALL treatment trials. Data on relapse and outcomes post relapse were available on these patients.

### Statistical analyses

Among the eligible patients enrolled on 12 frontline studies, EFS from date of initial diagnosis was calculated for each study, with events defined as induction failure, relapse, second malignant neoplasm, or death of any cause, whichever occurred first. Patient and disease characteristics at initial diagnosis were compared between patients who relapsed and those who did not using Pearson’s chi-squared tests. Among patients who relapsed, OS post-relapse was defined as the time between date of first relapse and date of death; patients alive at last follow-up were censored. Data cutoff date was June 30, 2021. The EFS or OS probabilities were calculated using the Kaplan–Meier method with Greenwood standard errors. Analyses of OS post-relapse in relation to patient and disease characteristics were based on the logrank tests and univariate and multivariable Cox regression models [27, 28]. Analyses were performed for B-ALL, T-ALL, and infant ALL separately. All *p*-values reported are two-sided. A *p* ≤ 0.05 was considered statistically significant. Statistical analyses were performed using STATA software [29].

## Results

Between June 1996 and July 2014, 16,115 patients enrolled on the 12 frontline clinical trials and 2053 (12.7%) relapsed (Table 1). Five-year EFS rates ranged from 45% to 91% depending upon the study population within each trial. For the entire relapse cohort, the median time-to-relapse was 31.0 months. Median follow-up from initial diagnosis was 121.7 months, and from first relapse, 70.5 months.

Most patients (13,771, 85.5%) had B-ALL, of which 12.5% relapsed (*n* = 1715) (Table 2). A significantly higher percentage of patients with National Cancer Institute (NCI) high-risk (HR) B-ALL relapsed (754/3974; 19%), compared to patients with NCI standard-risk (SR) B-ALL (961/9797; 9.8%; *p* < 0.0001). Among 2019 patients with T-ALL, 227 relapsed (11.2%) and of 325 infants, 111 relapsed (34.2%).

**Table 2:** Clinical characteristics and survival of 16115 patients included in this report.

Features at Initial Diagnosis	No. eligible (% in 16115 patients)	5-year EFS/OS rates		Relapse status			Time-to-relapse^d^ in months Median (range)	5-year post-relapse OS ± SE^e^
		5-year EFS ± SE^a^	5-year OS ± SE^a^	No. of patients without relapse (%)^b^	No. of patients with relapse (%)^b^	*P* value^c^		
All Patients	16115 (100%)	83.5 ± 0.3%	91.3 ± 0.2%	14062 (87.3%)	2053 (12.7%)		31.0 (0.3–186.0)	48.9 ± 1.2%
Sex						<.0001		
Male	9132 (56.7%)	82.8 ± 0.4%	90.9 ± 0.3%	7877 (86.3%)	1255 (13.7%)		30.2 (0.3–186.0)	48.3 ± 1.5%
Female	6983 (43.3%)	84.6 ± 0.5%	91.8 ± 0.3%	6185 (88.6%)	798 (11.4%)		32.1 (0.7–177.6)	49.9 ± 1.9%
Age						<.0001		
<1 year	325 (2.0%)	48.0 ± 2.8%	56.6 ± 2.8%	214 (65.8%)	111 (34.2%)		13.9 (3.4–57.5)	21.5 ± 3.9%
1–9 years	12224 (75.9%)	87.4 ± 0.3%	94.6 ± 0.2%	10878 (89.0%)	1346 (11.0%)		33.5 (0.3–186.0)	57.9 ± 1.4%
10–15 years	2541 (15.8%)	75.8 ± 0.9%	84.7 ± 0.7%	2140 (84.2%)	401 (15.8%)		28.4 (0.3–122.7)	35.9 ± 2.5%
≥16 years	1025 (6.4%)	68.0 ± 1.5%	79.2 ± 1.3%	830 (81.0%)	195 (19.0%)		29.3 (1.2, 103.3)	28.8 ± 3.5%
Race/ethnicity						<.0001		
Hispanic of all races	3190 (19.8%)	79.9 ± 0.7%	88.7 ± 0.6%	2698 (84.6%)	492 (15.4%)		29.8 (0.9–176.0)	45.4 ± 2.3%
Non-Hispanic White	9584 (59.5%)	85.3 ± 0.4%	92.5 ± 0.3%	8437 (88.0%)	1147 (12.0%)		33.4 (0.3–186.0)	51.7 ± 1.6%
Non-Hispanic Black	1014 (6.3%)	80.3 ± 1.3%	89.3 ± 1.0%	869 (85.7%)	145 (14.3%)		25.3 (0.7–108.1)	48.0 ± 4.3%
Non-Hispanic Asian	639 (4.0%)	83.7 ± 1.5%	91.5 ± 1.2%	574 (89.8%)	65 (10.2%)		30.4 (1.2–111.5)	42.8 ± 6.4%
Non-Hispanic Other	176 (1.1%)	81.4 ± 3.0%	88.8 ± 2.4%	156 (88.6%)	20 (11.4%)		20.5 (3.4–84.0)	35.6 ± 12.0%
Other/Unknown	1512 (9.4%)	82.6 ± 1.0%	90.1 ± 0.8%	1328 (87.8%)	184 (12.2%)		26.9 (0.3–139.5)	45.4 ± 3.8%
WBC (per µL)						<.0001		
<50k	12992 (80.7%)	86.5 ± 0.3%	93.5 ± 0.2%	11570 (89.1%)	1422 (10.9%)		35.2 (0.3–186.0)	52.4 ± 1.4%
50–100k	1271 (7.9%)	77.6 ± 1.2%	88.0 ± 0.9%	1054 (82.9%)	217 (17.1%)		30.1 (3.4–137.4)	53.6 ± 3.5%
≥100k	1841 (11.4%)	67.3 ± 1.1%	78.4 ± 1.0%	1427 (77.5%)	414 (22.5%)		17.7 (0.3–139.5)	34.6 ± 2.4%
Unknown	11			11				
CNS status						<.0001		
CNS 1	13802 (86.6%)	85.0 ± 0.3%	92.4 ± 0.2%	12152 (88.0%)	1650 (12.0%)		33.2 (0.3–177.6)	50.1 ± 1.3%
CNS 2	1724 (10.8%)	74.9 ± 1.1%	85.3 ± 0.9%	1415 (82.1%)	309 (17.9%)		22.7 (0.5–186.0)	44.5 ± 2.9%
CNS 3	405 (2.5%)	69.1 ± 2.4%	78.4 ± 2.1%	324 (80.0%)	81 (20.0%)		13.3 (3.3–92.7)	41.4 ± 5.6%
Unknown	184			171	13			
Testicular involvement						0.39		
No	8910 (99.1%)	82.9 ± 0.4%	91.0 ± 0.3%	7696 (86.4%)	1214 (13.6%)		30.2 (0.3–186.0)	48.5 ± 1.5%
Yes	83 (0.9%)	78.5 ± 4.8%	91.0 ± 3.3%	69 (83.1%)	14 (16.9%)		37.9 (8.7–71.6)	51.9 ± 14.3%
Unknown/NA	7122			6297	825			
Immunophenotype						<.0001		
B-Lineage (non-infants)	13771 (85.5%)	84.7 ± 0.3%	92.6 ± 0.2%	12056 (87.5%)	1715 (12.5%)		34.6 (1.7–186.0)	52.5 ± 1.3%
T-Lineage	2019 (12.5%)	81.9 ± 0.9%	87.9 ± 0.7%	1792 (88.8%)	227 (11.2%)		13.8 (0.3–132.5)	35.5 ± 3.3%
Infants (at initial dx)	325 (2.0%)	48.0 ± 2.8%	56.6 ± 2.8%	214 (65.8%)	111 (34.2%)		13.9 (3.4–57.5)	21.5 ± 3.9%
NCI risk group ^f^						<.0001		
Standard risk	9797 (71.1%)	89.2 ± 0.3%	95.7 ± 0.2%	8836 (90.2%)	961 (9.8%)		37.0 (2.1–186.0)	59.5 ± 1.7%
High risk	3974 (28.9%)	73.5 ± 0.7%	85.1 ± 0.6%	3220 (81.0%)	754 (19.0%)		31.6 (1.7–139.5)	43.5 ± 1.9%
POG/CCG vs. COG						<.0001		
POG/CCG	5674 (35.2%)	83.9 ± 0.5%	92.3 ± 0.4%	4852 (85.5%)	822 (14.5%)		30.5 (1.1, 186.0)	49.2 ± 1.8%
COG	10441 (64.8%)	83.4 ± 0.4%	90.7 ± 0.3%	9210 (88.2%)	1231 (11.8%)		31.5 (0.3, 169.4)	48.7 ± 1.5%
Induction Day 29 MRD^f^						<.0001		
<0.01%^h^	7093 (63.6%)	90.6 ± 0.4%	95.7 ± 0.3%	6506 (91.7%)	587 (8.3%)		35.7 (2.2, 162.5)	58.5 ± 2.1%
0.01–0.099%	2715 (24.4%)	83.6 ± 0.7%	94.3 ± 0.5%	2245 (82.7%)	470 (17.3%)		35.6 (2.3, 186.0)	57.6 ± 2.5%
0.1–0.99%	846 (7.6%)	68.2 ± 1.6%	83.1 ± 1.3%	597 (70.6%)	249 (29.4%)		35.5 (1.8, 176.0)	42.2 ± 3.3%
≥1.0%	491 (4.4%)	37.1 ± 2.3%	67.9 ± 2.2%	368 (74.9%)	123 (25.1%)		28.9 (1.7, 144.0)	28.8 ± 4.2%
Unknown	2626			2340	286			

*CCG* Children’s Cancer Group, *CNS* Central Nervous System, *COG* Children’s Oncology Group, *EFS* Event-free Survival, *MRD* Minimal Residual Disease, *NCI* National Cancer Institute, *OS* Overall Survival, *POG* Pediatric Oncology Group, *SE* Standard Error, *WBC* White Blood Cell Count.

^a^5-year EFS or OS of patients in each group from date of study enrollment. All patient/disease characteristic variables listed in the table were significantly associated with patients’ EFS or OS outcomes at *p* ≤ 0.003, except for testicular involvement (*p* values for its association with EFS and OS were 0.002 and 0.042, respectively) and POG/CCG vs. COG trials (*p* values for its association with EFS and OS were 0.042 and <0.001, respectively).

^b^Percent of total eligible patients in this row provided in the second column of the table.

^c^There was a statistically significant association between the proportion of patients having a relapse vs. each of the patient/disease characteristic variables included in the table (*p* < 0.0001), except with testicular involvement at initial diagnosis.

^d^Time from initial diagnosis to relapse among relapsed patients (time in months).

^e^5-year post-relapse OS among relapsed patients.

^f^Non-infant B-lineage only.

^g^National Cancer Institute (NCI)–Rome risk criteria standard. Standard Risk: age 1 through 9.99 years and diagnostic WBC count < 50,000/ml. High Risk: age ≥10 years or age 1 through 9.99 years with diagnostic WBC count ≥50,000/ml.

^h^Included 87 patients who whose MRD were “negative” with assay of sensitivity of only 1/1000.

### Relapse characteristics

**Fig. 1:**
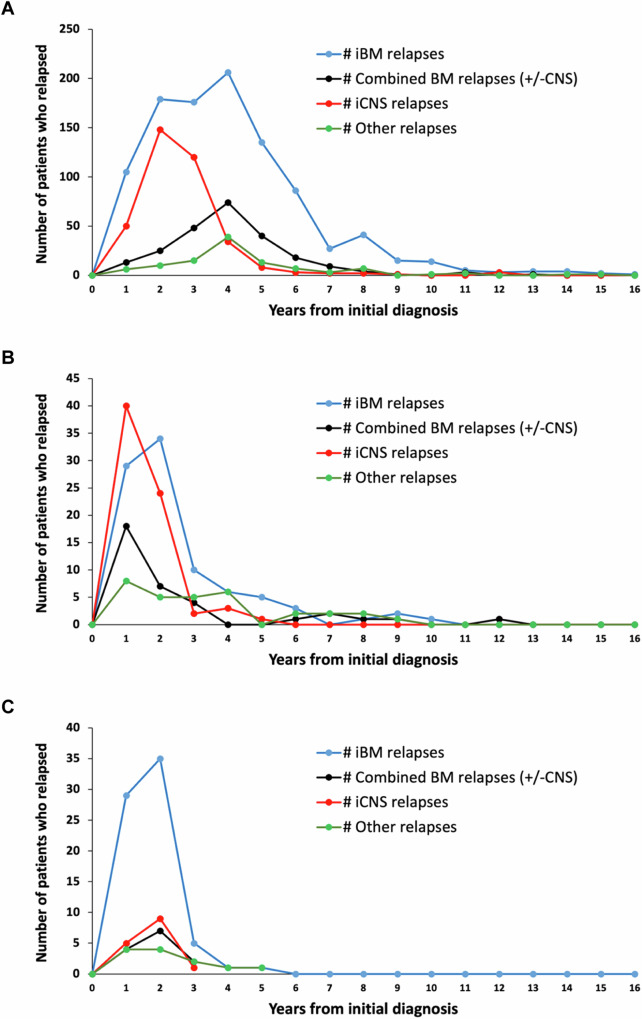
Time and site of relapse. Patterns of first relapse differed between (**A**) B-ALL, (**B**) T-ALL, (**C**) Infant ALL. iBM isolated bone marrow, BM bone marrow, CNS central nervous system, iCNS isolated central nervous system.

Characteristics of all patients with and without relapse are presented in Table 2. By univariate analysis relapse rate was significantly higher for males, as well as infants and patients aged ≥10 years at initial diagnosis, with incremental increased risk in patients ≥16 years. Patients of Hispanic ethnicity of all races, and those with WBC ≥ 50,000/uL or CNS 2/3 status at initial diagnosis also had significantly increased risk of relapse. The distribution of relapse site and time differed by immunophenotype and is detailed in Fig. 1 and Supplementary Table 1. Isolated testicular relapse was exceedingly rare (*n* = 66; 0.7%).

#### B-ALL

Patients with B-ALL had a median time-to-relapse of 34.6 months (range 1.7–186); significantly longer for NCI SR B-ALL compared to HR B-ALL (*p* < 0.0001) (37 months vs 31.6 months, Table 2). Approximately 16% of B-ALL relapses occurred >5 years from diagnosis and 1.7% >10 years (Fig. 1A). Patients with SR B-ALL accounted for over half of the B-ALL relapse cohort (961/1715). Patients with B-ALL most commonly experienced isolated bone marrow (iBM) relapses (58.7%); with any bone marrow (BM) involvement 72.5%, isolated CNS (iCNS) relapses 21.7%; and overall CNS involvement at relapse 32.9%. CNS relapses occurred earlier than BM relapses (Fig. 1A, Supplementary Table 1). Of 371 iCNS relapses, 113 occurred <18 months (30.5%), 205 (55.3%) 18- < 36 months and only 53 (14.3%) occurred ≥36 months. Patients with end-induction MRD ≥ 0.01% had increased risk of relapse (*p* < 0.0001).

Karyotype and FISH data for seven common cytogenetic subtypes were available at diagnosis for 10,094 of 13,771 patients with B-ALL (73.3%); 6440 patients were positive for one subtype; 3654 patients were negative for all seven subtypes, classified here as B-ALL, other. Complete cytogenetic data were not available for 3677 patients. Time-to-relapse differed among cytogenetic subtypes (Table 3). Patients with favorable cytogenetics: Trisomy 4 + 10 and *ETV6::RUNX1* relapsed later, with a median time-to-relapse of 43 months for both subtypes. They comprised 52% of newly diagnosed patients and 30% (391/1307) of the B-ALL relapse cohort. Patients with iAMP21 also had a long median time-to-relapse (43.6 months). Those with unfavorable subtypes, i.e., hypodiploid and *KMT2A*-rearranged (*KMT2A*-R), had very short time-to-relapse (12.5 and 18 months respectively). Patients with *TCF3::PBX1* also relapsed early, with median time-to-relapse 15.8 months.

**Table 3:** Impact of cytogenetics on time and site of relapse, and overall survival post relapse among non-infant B-ALL patients.

Cytogenetics^a^	No. of patients	No. of relapses (%)	5-yr from diagnosis EFS ± SE	Median time-to-relapse (months)	iBM relapse *N* (%)	Any BM relapse *N* (%)	iCNS relapse *N* (%)	Any CNS relapse N (%)	5-yr post relapse OS ± SE
Trisomy 4 + 10	2677	178 (6.6%)	92.6 ± 0.5%	43.3	108 (60.7%)	134 (75.3%)	31 (17.4%)	52 (29.2%)	70.2 ± 3.6%
*ETV6::RUNX1*	2555	213 (8.3%)	91.7 ± 0.6%	43.0	111 (52.1%)	151 (70.9%)	48 (22.5%)	77 (36.2%)	74.4 ± 3.1%
*KMT2A-R*	195	39 (20.0%)	70.2 ± 3.4%	18.0	23 (59.0%)	26 (66.7%)	6 (15.4%)	7 (17.9%)	31.9 ± 7.7%
*TCF3::PBX1*	414	57 (13.8%)	83.1 ± 1.9%	15.8	28 (49.1%)	31 (54.4%)	22 (38.6%)	25 (43.9%)	36.8 ± 6.6%
iAMP21	163	50 (30.7%)	68.5 ± 3.7%	43.6	31 (62.0%)	41 (82.0%)	8 (16.0%)	18 (36.0%)	48.2 ± 7.7%
*BCR::ABL1*	258	65 (25.2%)	52.4 ± 3.7%	35.3	52 (80.0%)	55 (84.6%)	8 (12.3%)	10 (15.4%)	47.2 ± 6.6%
Hypodiploid	178	36 (20.2%)	56.6 ± 4.1%	12.5	33 (91.7%)	34 (94.4%)	1 (2.8%)	2 (5.6%)	14.2 ± 6.1%
B-ALL, other	3654	669 (18.3%)	77.3 ± 0.7%	32.5	377 (56.4%)	472 (70.6%)	161 (24.1%)	246 (36.8%)	48.2 ± 2.0%

*ALL* Acute Lymphoblastic Leukemia, *BM* Bone Marrow, *CNS* Central Nervous System, *EFS* Event-free Survival, *i* Isolated, *OS* Overall Survival, *SE* Standard Error.

^a^Among patients included in the table, 15 patients had two cytogenetic features. For these patients, the higher risk cytogenetic feature was used to group them in this table.

Sites of relapse relative to cytogenetic subtype are detailed in Table 3. The proportion of patients with iBM relapse was highest for those with hypodiploidy (91.7%) and *BCR::ABL1* (80%), while CNS involvement at relapse, either isolated or combined, was most frequent in patients with *TCF3::PBX1* (43.9%).

#### T-ALL

In stark contrast to B-ALL, patients with T-ALL had a median time-to-relapse of 13.8 months (Table 2), the majority of whom (64.8%) relapsed <18 months from diagnosis and 82% relapsed <36 months from diagnosis. Relapse >5 years from diagnosis was less frequent than B-ALL (8.8%). The rate of iBM relapse in patients with T-ALL was 40.8%, any BM involvement 56.5%, iCNS relapse 31.4% and any CNS involvement at relapse 47.1% (Fig. 1B, Supplementary Table 1). Over 80% of iCNS relapses occurred <18 months from diagnosis. iCNS relapses accounted for 67.9%, 38.6%, and 21.1% of relapses that occurred in patients with CNS3, CNS2 and CNS1 at initial diagnosis respectively, demonstrating that burden of CNS disease at initial diagnosis was associated with iCNS site at relapse (Supplementary Table 2).

#### Infant ALL

Similar to patients with T-ALL, infants relapsed early with median time-to-relapse of 13.9 months (Table 2). Only 3.6% (4/111) infants who relapsed did so more than 36 months from diagnosis (Fig. 1C, Supplementary Table 1). BM was the most common site involved at relapse (77.8%) while 23.1% had any CNS involvement. Of 111 infants who relapsed, 98 were *KMT2A*-R and 13 were *KMT2A-*germline. Median time-to-relapse was 13.1 months (3.4–57.5) for *KMT2A-*R vs. 16.4 months (13.3–45.4) for *KMT2A*-germline infants (*p* = 0.009).

### Survival post-relapse

**Fig. 2:**
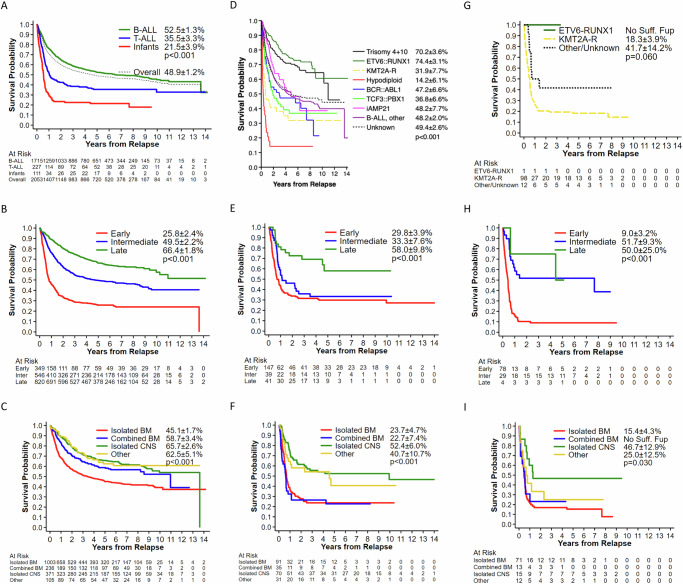
Overall survival post first relapse. Kaplan–Meier estimates of overall survival rates post relapse based on (**A**) mmunophenotype, (**B**) B-ALL time to relapse, (**C**) B-ALL site of relapse, (**D**) B-ALL cytogenetic subtype, (**E**) T-ALL time to relapse, (**F**) T-ALL site of relapse, (**G**) Infant ALL cytogenetic subtype, (**H**) Infant ALL time to relapse, (**I**) Infant ALL site of relapse.

Post-relapse 5-year OS was 48.9 ± 1.2% for all patients, 52.5 ± 1.3% for B-ALL, 35.5 ± 3.3% for T-ALL, and 21.5 ± 3.9% for infants (*p* < 0.001, Table 2, Fig. 2A). Enrollment on earlier era clinical trials (POG/CCG 1996–2006) did not show significantly different OS post-relapse (49.2 ± 1.8%) when compared to more contemporary COG trials conducted 2004–2014 (48.7 ± 1.5%) (Table 2).

#### B-ALL

Five-year post-relapse OS rates by patient and disease characteristics are presented in Supplementary Table 3, with univariate and multivariable analyses in Table 4. Variables that were significant by univariate analysis included time-to-relapse, relapse site, age and WBC at initial diagnosis, race/ethnicity, end-induction MRD at frontline therapy and age at relapse (all *p* ≤ 0.003). Longer time-to-relapse was associated with better OS post-relapse; 66.4 ± 1.8% for late relapse, 49.5 ± 2.2% for intermediate, and 25.8 ± 2.4% for early relapses (Fig. 2B, Supplementary Table 3). For relapse site, OS post-relapse was 45.1 ± 1.7% for iBM relapse, 58.7 ± 3.4% for combined BM relapse and 65.7 ± 2.6% for iCNS relapse (Fig. 2C, Supplementary Table 3). End-induction MRD ≥0.1% after frontline therapy was associated with inferior OS post-relapse: 58.2 ± 1.6% for patients with end-induction MRD < 0.1%, 37.7 ± 2.6% for those with MRD ≥ 0.1%; *p* < 0.005). All clinical factors remained significant in multivariable analysis, except for WBC at initial diagnosis and race/ethnicity as a group, although OS post-relapse for Non-Hispanic White patients remained significantly better than Hispanics of all races (*p* = 0.006).

**Table 4:** Univariate and multivariable analyses of risk factors on overall survival post relapse.

Variables	B-ALL				T-ALL				Infant ALL			
	univariate		multivariable		univariate		multivariable		univariate		multivariable	
	HR (95% CI)	*p*	HR (95% CI)	*p*	HR (95% CI)	*p*	HR (95% CI)	*p*	HR (95% CI)	*p*	HR (95% CI)	*p*
Time to relapse		<0.001		<0.001		<0.001		<0.001		<0.001		<0.001
Early (<18mo)	1		1		1		1		1		1	
Intermediate (18–36mo)	0.46 (0.39–0.55)		0.47 (0.40–0.57)		0.74 (0.48–1.13)		0.53 (0.34–0.85)		0.27 (0.15–0.48)		0.16 (0.08–0.31)	
Late (≥36mo)	0.26 (0.22–0.31)		0.21 (0.17–0.26)		0.33 (0.18–0.59)		0.23 (0.12–0.43)		0.23 (0.06–0.94)		0.16 (0.03–0.71)	
Relapse site		<0.001		<0.001		<0.001		<0.001		0.016		0.002
Isolated BM	1		1		1		1		1		1	
Combined BM ( ± CNS)	0.65 (0.52–0.81)		0.74 (0.60–0.93)		1.14 (0.73–1.80)		0.98 (0.59–1.61)		0.88 (0.45–1.71)		0.71 (0.34–1.49)	
Isolated CNS	0.53 (0.44–0.64)		0.32 (0.26–0.39)		0.41 (0.27–0.62)		0.27 (0.17–0.43)		0.35 (0.17–0.74)		0.23 (0.10–0.55)	
Other	0.51 (0.36–0.72)		0.49 (0.35–0.70)		0.50 (0.29–0.87)		0.49 (0.27–0.89)		0.66 (0.33–1.33)		1.46 (0.67–3.16)	
Sex		0.84		0.36		0.67		0.98		0.052		0.023
Male	1		1		1		1		1		1	
Female	0.99 (0.86–1.13)		0.93 (0.81–1.08)		0.92 (0.62–1.36)		0.99 (0.65–1.53)		1.52 (1.00–2.32)		1.81 (1.08–3.02)	
WBC at initial diagnosis (per µL)		<0.001		0.096		0.22		0.058		0.81		0.72
<50,000	1		1		1		1		1		1	
50,000–100,000	0.89 (0.69–1.14)		0.86 (0.66–1.11)		1.00 (0.56–1.79)		1.40 (0.73–2.71)		0.90 (0.44–1.83)		1.25 (0.55–2.80)	
≥100,000	1.60 (1.33–1.93)		1.18 (0.96–1.45)		1.34 (0.93–1.95)		1.69 (1.09–2.61)		1.08 (0.63–1.86)		0.94 (0.48–1.85)	
Age at initial diagnosis (non-infant)		<0.001		<0.001		0.002		0.002	Not applicable		Not applicable	
1–9 years	1		1		1		1					
10–15 years	1.92 (1.63–2.26)		1.59 (1.33–1.89)		1.98 (1.37–2.86)		2.00 (1.34–2.99)					
≥16 years	2.30 (1.88–2.83)		1.75 (1.40–2.18)		1.43 (0.85–2.43)		1.89 (1.07–3.35)					
Age at relapse^a^		<0.001	Excluded due to correlation with age at diagnosis			0.057	Excluded due to correlation with age at diagnosis			0.002	Excluded due to correlation with age at diagnosis	
<1 year	-								1			
1–9 years	1				1				0.37 (0.21-0.65)			
10–15 years	1.23 (1.04–1.45)				1.08 (0.74–1.59)							
≥16 years	1.61 (1.36–1.91)				1.70 (1.11–2.60)							
CNS status at initial dx		0.12		0.85		0.19		0.49		0.49		0.29
CNS 1	1		1		1		1		1		1	
CNS 2	1.14 (0.93–1.39)		0.98 (0.80–1.21)		0.94 (0.61–1.44)		0.96 (0.59–1.57)		0.83 (0.52–1.32)		1.04 (0.60–1.80)	
CNS 3	1.48 (0.96–2.29)		0.88 (0.56–1.38)		0.61 (0.35–1.07)		0.72 (0.39–1.32)		1.18 (0.65–2.14)		1.77 (0.85–3.65)	
Race/Ethnicity		0.003		0.13		0.74		0.46		0.68		0.025
Non-Hispanic White	1		1		1		1		1		1	
Hispanic of all races	1.39 (1.19–1.63)		1.26 (1.07–1.49)		0.87 (0.48–1.57)		0.91 (0.48–1.71)		0.72 (0.39–1.35)		0.52 (0.25–1.09)	
Non-Hispanic Black	1.28 (0.98–1.67)		1.21 (0.92–1.60)		0.88 (0.49–1.59)		0.71 (0.38–1.34)		0.52 (0.21–1.31)		0.23 (0.08–0.64)	
Non-Hispanic Asian	1.25 (0.84–1.85)		1.12 (0.75–1.68)		1.47 (0.59–3.64)		2.04 (0.78–5.35)		0.79 (0.31–1.96)		1.20 (0.43–3.34)	
Non-Hispanic Other	1.34 (0.66–2.69)		1.04 (0.51–2.12)		1.03 (0.14–7.40)		3.15 (0.35–28.61)		0.88 (0.28–2.82)		1.68 (0.47–6.03)	
Other/Unknown	1.03 (0.77–1.36)		1.04 (0.78–1.38)		1.26 (0.85–1.86)		1.07 (0.70–1.63)		0.82 (0.35–1.91)		0.69 (0.29–1.66)	
NCI risk group^b^		<0.001	Excluded due to correlation with age and WBC at diagnosis		Not assessed				Not assessed			
Standard risk	1											
High risk	1.67 (1.45–1.91)											
Induction Day 29 MRD^b^		<0.001		0.010	Not assessed				Not assessed			
<0.01%^**c**^	1		1									
0.01–0.099%	1.02 (0.85–1.23)		0.98 (0.81–1.19)									
0.1–0.99%	1.54 (1.26–1.90)		1.20 (0.97–1.49)									
≥1.0%	2.22 (1.74–2.84)		1.41 (1.09–1.84)									
Unknown	1.22 (0.99–1.50)		1.34 (1.02–1.75)									
Trisomy 4 + 10^b^		<0.001		0.004	Not applicable				Not applicable			
Negative	1		1									
Positive	0.54 (0.41–0.72)		0.66 (0.50–0.88)									
ETV6::RUNX1^b^		<0.001		0.001	Not applicable				Not applicable			
Negative	1		1									
Positive	0.42 (0.32–0.54)		0.62 (0.46–0.82)									
KMT2A-R^b^		<0.001		0.050	Not applicable					0.012		0.62
Negative	1		1						1		1	
Positive	2.04 (1.38–3.02)		1.52 (1.00–2.29)						2.40 (1.11–5.20)		1.24 (0.52–2.94)	
Hypodiploid^b^		<0.001		0.002	Not applicable				Not applicable			
Negative	1		1									
Positive	4.84 (3.33–7.03)		1.85 (1.25–2.75)									
BCR::ABL1^b^		0.044	Not significant; Excluded^*d*^		Not applicable				Not applicable			
Negative	1											
Positive	1.42 (1.01–1.99)											
TCF3::PBX1^b^		0.029	Not significant; Excluded^d^		Not applicable				Not applicable			
Negative	1											
Positive	1.46 (1.04–2.05)											
iAMP21^b^		0.99	Not significant; Excluded^d^		Not applicable				Not applicable			
No	1											
Yes	1.00 (0.67–1.50)											
B-ALL, other^b^		<0.001	Excluded due to correlation with the above cytogenetic variables		Not applicable				Not applicable			
No	1											
Yes	1.32 (1.12–1.55)											

*ALL* Acute Lymphoblastic Leukemia, *BM* Bone Marrow, *CI* Confidence Interval, *CNS* Central Nervous System, *HR* Hazard Ratio, *MRD* Minimal Residual Disease, *NCI* National Cancer Institute, *WBC* White Blood Cell Count.

^a^Age at relapse was associated with OS post relapse in univariate analysis and was excluded from multivariable analysis due to correlation with age at diagnosis. A sensitivity multivariable analysis (not reported) that included age at relapse did not render a significant *p* value for this variable.

^b^Characteristics at original diagnosis.

^c^Included 11 patients with negative MRD with assay of sensitivity of 1/1000.

^d^No significant association was found between the three cytogenetic variables and survival post relapse after adjusting for other variables. Due to small sample size in subcategories of the cytogenetic variables, the three cytogenetic variables were excluded from the multivariable model.

Post-relapse OS was significantly associated with cytogenetic subtype (*p* < 0.001, Table 3, Fig. 2D). Similar to de novo B-ALL, cytogenetic subtypes with the best OS post-relapse were *ETV6::RUNX1* and Trisomy 4 + 10 with 5-year OS of 74.4 ± 3.1% and 70.2 ± 3.6% respectively, while those with the worst outcomes were hypodiploid (14.2 ± 6.1%), and *KMT2A-R* (31.9 ± 7.7%). For the other subtypes, 5-year OS post-relapse ranged from 36.8–48.2%. All cytogenetic features except iAMP21 were significantly associated with OS post-relapse in B-ALL in univariate analysis. Only Trisomy 4 + 10 (*p* = 0.004), *ETV6::RUNX1* (*p* = 0.001)*, KMT2A*-R (*p* = 0.050) and hypodiploid (*p* = 0.002) remained significant after adjusting for time-to-relapse, relapse site(s) and other patient and disease characteristics (Table 4).

#### T-ALL

For patients with T-ALL, time-to-relapse, relapse site, and age at initial diagnosis were significantly associated with OS post-relapse in univariate and multivariable analyses (all *p* ≤ 0.002; Table 4). Unlike B-ALL, race and ethnicity were not significant for survival post-relapse (Table 4, Supplementary Table 3). Five-year OS post-relapse was highest for patients with late relapse (58 ± 9.8%) but was equally poor for T-ALL patients with intermediate (33 ± 7.6%) or early (29.8 ± 3.9%) relapse (Fig. 2E). For site of relapse, iBM and combined BM relapse had similar 5-year OS post-relapse (23.7 ± 4.7% and 22.7 ± 7.4%, respectively) compared to 52.4 ± 6% for iCNS (Fig. 2F).

#### Infant ALL

Time-to-relapse (*p* < 0.001) and relapse site (*p* = 0.002) were significantly associated with OS post-relapse in multivariable analysis (Table 4). In addition, female infants had higher risk of death compared to males (*p* = 0.023). Infants with early relapses <18 months rarely survived (9.0 ± 3.2% 5-year OS post-relapse), but OS post-relapse was 51.1 ± 8.8% for infants with relapses ≥18 months (Fig. 2H). Infants with iBM involvement at relapse had significantly worse OS than those with combined BM or iCNS relapses (Fig. 2I). No infant with age at relapse <1 year survived (Supplementary Table 3).

Five-year OS post-relapse was 18.3 ± 3.9% for *KMT2A*-R versus 46.2 ± 13.8% for *KMT2A*-germline (*p* = 0.012, Table 3, Supplementary Table 3), but *KMT2A*-R was not significantly associated with OS post-relapse after adjusting for time-to-relapse, relapse site and other characteristics (*p* = 0.62).

## Discussion

We analyzed the largest dataset to date of pediatric, adolescent and young adult patients with B-ALL, T-ALL, and infant ALL and describe clinical features at first relapse, as well as prognostic indicators for survival after relapse. In comparison to the previous COG report [7], relapse rates decreased from 20.5% to 12.7%. Patients with relapsed B-ALL had significant improvement in 5-year OS post-relapse (37.2 ± 21% prior vs. 52.5 ± 1.3% current, *p* < 0.001); more apparent in NCI HR-ALL (22.6 ± 2.1% prior vs. 43.5 ± 1.9% current, *p* < 0.001) compared to SR-ALL (50.4 ± 2.4% prior vs. 59.5 ± 1.7% current, *p* < 0.01). Patients with T-ALL had improved OS post-relapse of 35.5 ± 3.3%, compared to 23.0 ± 4.0% in the prior COG report (*p* < 0.05), while OS post-relapse for infants remained dismal at approximately 20% in both cohorts.

When assessed by immunophenotype, time and site of relapse are distinct. Half of B-ALL relapses occur ≥36 months post-diagnosis and over 70% involve the bone marrow, whereas 82% of T-ALL relapses occur within 36 months and the CNS is involved in almost half of cases. The majority (67.9%) of patients with iCNS relapse of T-ALL had CNS3 disease at initial diagnosis, despite receiving therapeutic cranial irradiation on frontline regimens suggesting inadequate clearance of this sanctuary site. Though CNS relapses occur earlier than BM/combined relapses in both B and T-ALL they had significantly better OS post-relapse. Early relapse of B-ALL and T-ALL have significantly worse outcomes but T-ALL patients with intermediate relapses did not have the survival advantage seen with B-ALL, possibly accounting for the worse OS for T-ALL post-relapse.

Beyond known risk factors, i.e., time-to-relapse, site of relapse, immunophenotype and age <1 year or ≥10 years [7, 10, 11], we identified additional risk factors that independently predict for worse outcomes in relapsed B-ALL. End-induction MRD ≥ 0.01% during frontline therapy increased risk of relapse, but a higher MRD threshold of ≥0.1% at end-induction was associated with worse OS post-relapse on multivariable analyses. End-induction MRD ≥ 1% had an even shorter time to relapse and 5-year OS post-relapse. We also demonstrate that cytogenetic subtype in B-ALL is an important prognostic factor at relapse and is closely linked to time-to-relapse, substantiating that biology drives clinical behavior. Interestingly, patients with *TCF3::PBX1*, considered cytogenetically neutral at initial diagnosis, had short time-to-relapse (median 15.8 months) and inferior OS post-relapse (36.8±6.6%) while patients with iAMP21, traditionally considered a high-risk subtype at initial diagnosis, had one of the longest time-to-relapse (median 43.6 months) with a 48.2 ± 7.7% OS post-relapse. These findings indicate that tumor biology associated with cytogenetic subtypes alters clonal dynamics and the emergence of subclones with varying responses to retrieval therapy [30–32]. Further investigation by next generation testing including single cell sequencing of diagnosis, end-induction MRD and relapse blasts will likely provide insights into the dynamics of resistance and relapse [33]. In multivariable analysis, Trisomy 4 + 10, *ETV6::RUNX1, KMT2A-*R and hypodiploidy retained their independent significant impact on outcome after adjusting for time-to-relapse and other variables. It remains to be seen if risk stratification algorithms at relapse can be refined by incorporating cytogenetic variables similar to initial diagnosis. More recently described cytogenetic subtypes with prognostic impact, such as Ph-like ALL, or secondary lesions such as TP53 and RAS mutations were not tested in this cohort but are being considered for new risk stratification algorithms [10, 34].

Our analysis by race and ethnicity was limited by the use of individual institutional submission but did show that Hispanic patients with B-ALL of all races were more likely to have worse outcomes post-relapse than non-Hispanic White patients. In addition to race and ethnicity, it is imperative going forward that clinical trials collect comprehensive data on non-medical social determinants of health which also likely influences outcomes. Intervention in these domains in populations at risk will be required to improve survival post relapse.

Infant ALL remains one of the greatest challenges; sequential studies have failed to demonstrate any improvement in survival post-relapse [17, 23]. Infants who relapse <18 months have a dismal 9.0 ± 3.2% post-relapse OS and no infant who relapsed while still <1 year of age survived. With our small sample size of *KMT2A*-germline patients (*n* = 13), the analysis assessing independent contribution of *KMT2A-*R in post-relapse outcomes in infants lacked statistical power. We were able to identify some infant ALL populations who are more salvageable, including those who relapse ≥18 months from diagnosis, infants with iCNS relapse and infants without *KMT2A*-R (5-yr OS 51.1 ± 8.8%, 46.7 ± 12.9% and 46.2 ± 13.8%, respectively). Further intensification of therapy for infants is challenging due to toxicity and targeted therapies to date have not showed benefit [23]. Early data combining blinatumomab with chemotherapy is promising for newly diagnosed *KMT2A-*R infants [35].

The significance of some of the findings from this study could be altered by more modern frontline therapy; including changes in CNS-directed therapy which evolved during the study period [18]. To assess this concern, we performed an analysis by “era treated” comparing the earlier CCG/POG trials (1996–2006) versus more contemporary COG trials (2004–2014) and did not detect outcome differences seen between the two groups post-relapse. A similar result was noted in the prior COG analysis wherein historical era of initial treatment, as a surrogate marker of therapy intensity, did not predict for OS post-relapse [2]. In addition, the CCG-1961 trial randomizing patients with HR B-ALL and T-ALL to intensified versus standard therapy post-Induction, showed no difference in post-relapse survival on the more intensive therapy arm [36].

A major limitation to these data is the lack of information about relapse therapy received, including transplant utilization; as well as MRD response to relapse re-induction therapy. We also lack additional information on post-relapse events such as subsequent relapse or death due to toxicity during chemotherapy or transplant. It is presumed most patients would have received intensive first relapse chemotherapy ± radiation to involved extramedullary sites, with or without a consolidative stem cell transplant. Another limitation is that our cohort only includes patients who were enrolled on front line pediatric therapy trials during time periods they were open. However, the wide catchment area of the over 200 COG treatment centers and the size of our cohort provides a broad representation of all children with ALL.

Although overall survival post-relapse rates appear to be improved when compared to our prior analyses, our data are unlikely to reflect the current revolution in immunotherapy and targeted therapy [13, 14, 37]. Only a minority of patients in this cohort enrolled on COG first ALL relapse trials involving investigational immunotherapies and targeted therapies. The use of tyrosine kinase inhibitors (TKIs) for Ph+ ALL increased significantly during this time period with pediatric de novo ALL trials incorporating TKIs into therapy as of the early 2000’s [38, 39]. Post-induction, 33% of our Ph+ ALL cohort enrolled on TKI containing COG frontline therapy trials (AALL0031 and AALL0622) but due to small patient numbers it was not possible to decipher if TKI use upfront impacted OS rate post relapse. It remains to be seen if newer interventions for relapsed ALL, including CAR T-cell immunotherapy, will improve long-term survival. The results reported herein will be valuable as a benchmark to compare the impact of immunotherapy and small molecule targeted therapy in improving survival post relapse [40, 41].

For patients with truly dismal outcomes post-relapse: infants, adolescents ≥16 years, T-ALL relapses involving the BM, and hypodiploid ALL, it would be prudent to consider alternatives to conventional cytotoxic chemotherapy at relapse. In a separate analysis patients with Down Syndrome on frontline COG trials also fit into the dismal outcomes category with a post-relapse 5-year OS of 14.3 ± 8.9% [26]. The COG current first relapse ALL trial studying blinatumomab and nivolumab with chemotherapy, AALL1821 (NCT04546399), now stratifies patient therapy based upon age ≥18 years, regardless of time-to-relapse given their poor survival outcomes and known increased toxicity in this population. International collaborations, developing combined pediatric and adult trails for the adolescent and young adult population and partnerships with industry to design trials of synergistic combinations of targeted therapies may overcome some of the challenges associated with clinical trials in smaller patient populations.

Despite these limitations, this large dataset has provided the opportunity to study various patient cohorts based on time to and site of relapse that including immunophenotype, cytogenetics, and race/ethnicity, which should inform future trial design and highlight populations for testing more novel therapy options.

## Supplementary information

Supplementary Tables 1–3

## Data Availability

De-identified data from each of the included Children’s Oncology Group trials are available upon request to the relevant trial committees. Requests for access to COG protocol research data should be sent to: datarequest@childrensoncologygroup.org.
