# Supplementary material for: Determinants of survival after first relapse of acute lymphoblastic leukemia: a Children’s Oncology Group study
Source: Leukemia. 2024 Sep 11;38(11):2382–94. doi: 10.1038/s41375-024-02395-4 (PMC11518984; doi:10.1038/s41375-024-02395-4)
Supplement: Supplementary file 1 — Supplementary Tables 1–3 [file 41375_2024_2395_MOESM1_ESM.docx]

**Supplemental Materials**

**Supplemental Table 1: Time and site of relapse by immunophenotype**

|  | **B-ALL** | | | | **T-ALL** | | | | **Infant ALL** | | | |
| --- | --- | --- | --- | --- | --- | --- | --- | --- | --- | --- | --- | --- |
|  |  | **Time to Relapse** | | |  | **Time to Relapse** | | |  | **Time to Relapse** | | |
|  | **Total**  **B-ALL** | **Early**  **(<18 months)** | **Intermediate (18-<36 months)** | **Late (≥36 months)** | **Total**  **T-ALL** | **Early (<18 months)** | **Intermediate (18-<36 months)** | **Late (≥36 months)** | **Total Infant** | **Early (<18 months)** | **Intermediate (18-<36 months)** | **Late (≥36 months)** |
| **Relapse Site(s)** | **n (%^a^)** | **n (%)** | **n (%)** | **n (%)** | **n (%)** | **n (%)** | **n (%)** | **n (%)** | **n (%)** | **n (%)** | **n (%)** | **n (%)** |
| **Isolated BM** | 1003 (58.7%) | 195 (56.0%) | 265  (48.5%) | 543 (66.7%) | 91 (40.8%) | 53  (36.6%) | 20  (54.1%) | 18 (43.9%) | 71 (65.7%) | 54 (71.1%) | 15  (53.6%) | 2  (50%) |
| **Combined BM (CNS+)** | 186 (10.9%) | 26 (7.5%) | 51  (9.3%) | 109 (13.4%) | 31 (13.9%) | 23  (15.9%) | 3  (8.1%) | 5 (12.2%) | 10 (9.3%) | 6  (7.9%) | 4  (14.3%) | 0 |
| **Combined BM (CNS-)** | 50 (2.9%) | 2  (0.6%) | 7  (1.3%) | 41 (5.0%) | 4 (1.8%) | 1  (0.7%) | 2  (5.4%) | 1  (2.4%) | 3 (2.8%) | 2  (2.6%) | 1  (3.6%) | 0 |
| **Isolated CNS** | 371 (21.7%) | 113 (32.5%) | 205  (37.5%) | 53 (6.5%) | 70 (31.4%) | 57 (39.3%) | 9  (24.3%) | 4  (9.8%) | 15 (13.9%) | 9 (11.8%) | 6  (21.4%) | 0 |
| **Isolated Testicular** | 58 (3.4%) | 3 (0.9%) | 9  (1.6%) | 46 (5.7%) | 2 (0.9%) | 1  (0.7%) | 0 | 1  (2.4%) | 6 (5.6%) | 4  (5.3%) | 1  (3.6%) | 1  (25%) |
| **Other extra-medullary (±CNS)** | 40 (2.3%) | 9 (2.6%) | 9  (1.6%) | 22 (2.7%) | 25 (11.2%) | 10 (6.9%) | 3  (8.1%) | 12 (29.3%) | 3 (2.8%) | 1  (1.3%) | 1  (3.6%) | 1  (25%) |
| **Unspecified** | 7 | 1 | 0 | 6 | 4 | 2 | 2 | 0 | 3 | 2 | 1 | 0 |
| **All Relapses** | 1715 | 349 | 546 | 820 | 227 | 147 | 39 | 41 | 111 | 78 | 29 | 4 |
| **Any BM involvement** | 1239 (72.5%) | 223 (64.1%) | 323  (59.2%) | 693 (85.1%) | 126 (56.5%) | 77 (53.1%) | 25  (67.6%) | 24 (58.5%) | 84 (77.8%) | 62 (81.6%) | 20  (71.4%) | 2 (50.0%) |
| **Any CNS involvement** | 562 (32.9%) | 141 (40.5%) | 257  (47.1%) | 164 (20.1%) | 105 (47.1%) | 83 (57.2%) | 13  (35.1%) | 9 (22.0%) | 25 (23.1%) | 15 (19.7%) | 10  (35.7%) | 0 |
| **Any Testicular involvement** | 100 (5.9%) | 3  (0.9%) | 16  (2.9%) | 81 (10.0%) | 4 (1.8%) | 1 (0.7%) | 2  (5.4%) | 1 (2.4%) | 9 (8.3%) | 6  (7.9%) | 2  (7.1%) | 1 (25.0%) |

Abbreviations: ALL, Acute Lymphoblastic Leukemia; BM, Bone Marrow; CNS, Central Nervous System

^a^ % represents column percentages.

**Supplemental Table 2: Patterns of relapse based on CNS status at diagnosis**

| **CNS status at initial diagnosis** | **B-ALL**  N (%^b^) | | | | **T-ALL**  N (%) | | | | **Infant ALL**  N (%) | | | |
| --- | --- | --- | --- | --- | --- | --- | --- | --- | --- | --- | --- | --- |
|  | Isolated CNS | Combined BM+CNS | Isolated BM | Other^a^ | Isolated CNS | Combined BM+CNS | Isolated BM | Other^a^ | Isolated CNS | Combined BM+CNS | Isolated BM | Other^a^ |
| CNS1 | 288  (19.9%) | 150  (10.4%) | 882  (60.9%) | 129  (8.9%) | 32  (21.1%) | 24  (15.8%) | 66  (43.4%) | 30  (19.7%) | 2  (4.1%) | 3  (6.1%) | 35  (71.4%) | 9  (18.4%) |
| CNS2 | 73  (32.9%) | 33 (14.9%) | 95  (42.8%) | 21  (9.5%) | 17  (38.6%) | 5  (11.4%) | 19  (43.2%) | 3  (6.8%) | 9  (20.9%) | 4  (9.3%) | 25  (58.1%) | 5  (11.6%) |
| CNS3 | 9  (25.7%) | 2  (5.7%) | 23  (65.7%) | 1  (2.9%) | 19  (67.9%) | 2  (7.1%) | 5  (17.9%) | 2  (7.1%) | 4  (22.2%) | 3  (16.7%) | 10  (55.6%) | 1  (5.6%) |

ALL, Acute Lymphoblastic Leukemia; BM, Bone Marrow, CNS, Central Nervous System

^a^ Other includes relapse sites of the following categories: combined BM without CNS involvement, isolated testicular, other extra-medullary ±CNS, and unspecified.

^b^ % represents row percentages.

**Supplemental Table 3: 5-year post relapse survival by patient and disease characteristics**

|  | **B-ALL** | | | **T-ALL** | | | **Infant ALL** | | |  |
| --- | --- | --- | --- | --- | --- | --- | --- | --- | --- | --- |
| ***Variables*** | **No. pts** | **No. deaths** | **5-year OS post relapse (**±**SE)** | **No. pts** | **No. deaths** | **5-year OS post relapse (**±**SE)** | **No. pts** | **No. deaths** | **5-year OS post relapse (**±**SE)** |  |
|  |  |  |  |  |  |  |  |  |  |  |
| ***Time to relapse*** |  |  |  |  |  |  |  |  |  |  |
| Early (<18mo) | 349 | 256 | 25.8±2.4% | 147 | 102 | 29.8±3.9% | 78 | 71 | 9.0±3.2% |  |
| Intermediate (18-<36mo) | 546 | 285 | 49.5±2.2% | 39 | 26 | 33.3±7.6% | 29 | 15 | 51.7±9.3% |  |
| Late (≥36mo) | 820 | 268 | 66.4±1.8% | 41 | 13 | 58.0±9.8% | 4 | 2 | 50.0±25.0% |  |
| ***Relapse site*** |  |  |  |  |  |  |  |  |  |  |
| Isolated BM | 1003 | 537 | 45.1±1.7% | 91 | 65 | 23.7±4.7% | 71 | 61 | 15.4±4.3% |  |
| Combined BM (±CNS) | 236 | 96 | 58.7±3.4% | 35 | 26 | 22.7±7.4% | 13 | 10 | Insufficient follow up |  |
| Isolated CNS | 371 | 140 | 65.7±2.6% | 70 | 34 | 52.4±6.0% | 15 | 8 | 46.7±12.9% |  |
| Other | 105 | 36 | 62.5±5.1% | 31 | 16 | 40.7±10.7% | 12 | 9 | 25.0±12.5% |  |
| ***Sex*** |  |  |  |  |  |  |  |  |  |  |
| Male | 1019 | 488 | 52.1±1.6% | 174 | 109 | 35.1±3.8% | 62 | 47 | 23.8±5.5% |  |
| Female | 696 | 321 | 53.2±2.0% | 53 | 32 | 36.8±7.0% | 49 | 41 | 18.4±5.5% |  |
| ***WBC at initial diagnosis (per µL)*** |  |  |  |  |  |  |  |  |  |  |
| <50k | 1326 | 605 | 53.6±1.4% | 75 | 42 | 40.3±6.1% | 21 | 17 | 19.1±8.6% |  |
| 50-100k | 169 | 69 | 58.4±4.0% | 28 | 16 | 42.0±9.5% | 20 | 14 | 30.0±10.3% |  |
| ≥100k | 220 | 135 | 41.5±3.4% | 124 | 83 | 30.9±4.3% | 70 | 57 | 19.6±4.8% |  |
| ***Age at initial diagnosis^a^*** |  |  |  |  |  |  |  |  |  |  |
| <1 year (Infants) | - | - | - | - | - | - | 111 | 88 | 21.5±3.9% |  |
| 1-9 years | 1202 | 489 | 59.7±1.5% | 144 | 78 | 43.0±4.3% | - | - | - |  |
| 10-15 years | 344 | 208 | 38.6±2.8% | 57 | 46 | 20.1±5.4% | - | - | - |  |
| ≥16 years | 169 | 112 | 28.8±3.8% | 26 | 17 | 30.0±9.7% | - | - | - |  |
| ***Age at relapse^b^*** |  |  |  |  |  |  |  |  |  |  |
| <1 year | - | - | - | - | - | - | 16 | 16 | 0% |  |
| 1-9 years | 875 | 381 | 57.3±1.7% | 111 | 65 | 39.9±4.7% | 95 | 72 | 25.1±4.5% |  |
| 10-15 years | 482 | 229 | 52.4±2.4% | 74 | 44 | 37.0±6.1% | - | - | - |  |
| ≥16 years | 358 | 199 | 39.8±2.8% | 42 | 32 | 19.6±6.7% | - | - | - |  |
| ***CNS status at initial diagnosis*** |  |  |  |  |  |  |  |  |  |  |
| CNS 1 | 1449 | 671 | 53.1±1.4% | 152 | 99 | 32.3±4.0% | 49 | 40 | 17.9±5.6% |  |
| CNS 2 | 222 | 112 | 49.7±3.5% | 44 | 27 | 34.6±7.6% | 43 | 32 | 27.9±6.8% |  |
| CNS 3 | 35 | 21 | 46.7±8.6% | 28 | 14 | 51.0±9.8% | 18 | 15 | 16.7±8.8% |  |
| Unknown | 9 | 5 | 44.4±16.6% | 3 | 1 | 66.7±27.2% | 1 | 1 | 0% |  |
| ***Race/ethnicity*** |  |  |  |  |  |  |  |  |  |  |
| Hispanic of all races | 452 | 242 | 46.2±2.4% | 24 | 13 | 43.7±10.4% | 16 | 12 | 25.0±10.8% |  |
| Non-Hispanic White | 957 | 418 | 55.7±1.7% | 120 | 71 | 38.2±4.7% | 70 | 57 | 20.0±4.8% |  |
| Non-Hispanic Black | 115 | 61 | 51.0±4.8% | 22 | 13 | 34.7±11.2% | 8 | 5 | 37.5±17.1% |  |
| Non-Hispanic Asian | 52 | 26 | 48.7±7.2% | 7 | 5 | Insufficient follow up | 6 | 5 | 0% |  |
| Non-Hispanic Other | 16 | 8 | 49.2±12.7% | 1 | 1 | 0% | 3 | 3 | 0% |  |
| Other/Unknown | 123 | 54 | 54.5±4.7% | 53 | 38 | 27.9±6.2% | 8 | 6 | 25.0±15.3% |  |
| ***NCI risk group (non-infant B-lineage only)*** |  |  |  | Not assessed | | | Not assessed | | |  |
| Standard risk | 961 | 390 | 59.5±1.7% |  |  |  |  |  |  |  |
| High risk | 754 | 419 | 43.5±1.9% |  |  |  |  |  |  |  |
| ***Day 29 MRD value*** |  |  |  | Not assessed | | | Not assessed | | |  |
| <0.01 %^c^ | 587 | 246 | 58.5±2.1% |  |  |  |  |  |  |  |
| 0.01–0.099 % | 470 | 197 | 57.6±2.5% |  |  |  |  |  |  |  |
| 0.1-0.99 % | 249 | 141 | 42.2±3.3% |  |  |  |  |  |  |  |
| ≥1.0 % | 123 | 87 | 28.8±4.2% |  |  |  |  |  |  |  |
| Unknown | 286 | 138 | 50.9±3.1% |  |  |  |  |  |  |  |
| ***Trisomy 4+10*** |  |  |  | Not applicable | | | Not applicable | | |  |
| Negative | 1299 | 639 | 50.4±1.5% |  |  |  |  |  |  |  |
| Positive | 180 | 55 | 70.4±3.6% |  |  |  |  |  |  |  |
| Unknown | 236 | 115 | 50.9±3.4% |  |  |  |  |  |  |  |
| ***ETV6::RUNX1*** |  |  |  | Not applicable | | | Not applicable | | |  |
|  |  |  |  |  |  |  |  |  |  |  |
| Negative | 1190 | 602 | 48.6±1.5% |  |  |  |  |  |  |  |
| Positive | 213 | 57 | 74.4±3.1% |  |  |  |  |  |  |  |
| Unknown | 312 | 150 | 52.4±2.9% |  |  |  |  |  |  |  |
| ***KMT2A-R*** |  |  |  | Not applicable | | |  |  |  |  |
| Negative | 1528 | 713 | 53.2±1.3% |  |  |  | 13 | 7 | 46.2±13.8% |  |
| Positive | 39 | 26 | 31.9±7.7% |  |  |  | 98 | 81 | 18.3±3.9% |  |
| Unknown | 148 | 70 | 51.6±4.3% |  |  |  |  |  |  |  |
| ***Hypodiploid*** |  |  |  | Not applicable | | | Not applicable | | |  |
| Negative | 1644 | 768 | 52.9±1.3% |  |  |  |  |  |  |  |
| Positive | 36 | 29 | 14.2±6.1% |  |  |  |  |  |  |  |
| Unknown | 35 | 12 | 70.2±7.9% |  |  |  |  |  |  |  |
| ***BCR::ABL1*** |  |  |  | Not applicable | | | Not applicable | | |  |
| Negative | 1617 | 760 | 52.6±1.3% |  |  |  |  |  |  |  |
| Positive | 65 | 35 | 47.2±6.6% |  |  |  |  |  |  |  |
| Unknown | 33 | 14 | 59.6±8.7% |  |  |  |  |  |  |  |
| ***TCF3::PBX1*** |  |  |  | Not applicable | | | Not applicable | | |  |
| Negative | 1387 | 652 | 53.0±1.4% |  |  |  |  |  |  |  |
| Positive | 57 | 35 | 36.8±6.6% |  |  |  |  |  |  |  |
| Unknown | 271 | 122 | 53.6±3.2% |  |  |  |  |  |  |  |
| ***iAMP21*** |  |  |  | Not applicable | | | Not applicable | | |  |
| No | 913 | 428 | 53.3±1.7% |  |  |  |  |  |  |  |
| Yes | 50 | 25 | 48.2±7.7% |  |  |  |  |  |  |  |
| Unknown | 752 | 356 | 51.7±1.9% |  |  |  |  |  |  |  |
| ***B-ALL, other*** |  |  |  | Not applicable | | | Not applicable | | |  |
| No | 638 | 262 | 59.1±2.0% |  |  |  |  |  |  |  |
| Yes | 669 | 342 | 48.2±2.0% |  |  |  |  |  |  |  |
| Unknown | 408 | 205 | 49.4±2.6% |  |  |  |  |  |  |  |

Abbreviations: ALL, Acute Lymphoblastic Leukemia; BM, Bone Marrow, CNS, Central Nervous System; OS, Overall Survival; pts, patients; SE, Standard Error; WBC, White Blood Cell Count

^a^ A total of 2053 patients (1715 B-ALL, 227 T-ALL, 111 Infant ALL) had a 1^st^ relapse and were included in this table, among whom median age at initial diagnosis was 5.9 years (range 0-30), (B-ALL 6.2 years, T-ALL 7.8 years, Infant ALL 0.42 years).

^b^ Median age at relapse: 9.4 years, range 0.4-32.9 years, (B-ALL 9.8 years, T-ALL 10.1 years, Infant ALL 1.67 years)

^c^ Included 11 patients with negative MRD with assay of sensitivity of 1/1000.
